# Thymol and menthol as anaesthetics for short transportation of zebrafish larva

**DOI:** 10.1007/s10695-025-01530-x

**Published:** 2025-07-30

**Authors:** Raquel S. F. Vieira, Carlos A. S. Venâncio, Luís M. Félix

**Affiliations:** 1 School of Life and Environmental Sciences, ECVA, University of Trás-Os-Montes and Alto Douro, UTAD, Vila Real, Portugal; 2 Department of Animal Science, School of Agrarian and Veterinary Sciences, ECAV, University of Trás-Os-Montes and Alto Douro, UTAD, Vila Real, Portugal; 3 Animal and Veterinary Research Centre, CECAV, University of Trás-Os-Montes and Alto Douro, UTAD, Vila Real, Portugal; 4 Centre for the Research and Technology of Agroenvironmental and Biological Sciences, CITAB, Inov4Agro, Universidadede Trás-os-Montes e Alto Douro, UTAD, Vila Real, Portugal

**Keywords:** Anaesthesia, Monoterpenes, Stress, Transport, Zebrafish larvae

## Abstract

Transportation-induced stress is a major concern in aquaculture, often causing physiological and behavioural disruptions in fish. To alleviate these effects, anaesthesia is commonly used, but the most widely used anaesthetics, MS-222 and eugenol, have been linked to adverse effects, and alternatives are lacking. This study evaluated the anaesthetic efficacy of thymol and menthol, in comparison with MS-222 and eugenol, under stress conditions induced by a simulated 4-h transportation of 96 hpf zebrafish larvae. Based on LC_50_ values, the transport simulation was conducted with 200 mg L^−1^ MS-222, 80 mg L^−1^ eugenol, 15 mg L^−1^ thymol, and 50 mg L^−1^ menthol. Parameters assessed included heart rate, biochemical markers, stress responses, metabolic rate, protein expression related to oxidative stress and cell death, and potential inflammation. No changes in cortisol levels were observed possibly reflecting negative feedback of the HPI axis. However, signs of stress were evident through increased metabolic rate and reduced ATPase activity. The use of anaesthetics reduced glucose spikes and lowered heart rate and ATPase activity suggesting a shift towards a hypometabolic state. In terms of oxidative stress, GST activity returned to baseline, but GSH levels were further reduced by anaesthetics. Anaesthetics increased SOD levels and decreased CAT activity, with menthol causing a significant rise in Nrf2. Despite changes in oxidative stress markers, no alterations in cellular damage or inflammation markers were found. While further studies are needed, thymol and menthol did not significantly disrupt physiological balance, supporting their potential use in aquaculture.

**Supplementary Information:**

The online version contains supplementary material available at 10.1007/s10695-025-01530-x.

## Introduction

Improving welfare practices in aquaculture is a growing need as there are procedures such as handling, weighing, reproduction, and transportation that can cause welfare disturbances and stress in animals (Ashley 2007; Barreto et al. 2021; Mancuso 2013). Thus, it is necessary to minimize the effects of these procedures and maintain animal comfort. For this, a practice increasingly implemented is the use of sedatives and anaesthetics with positive effects on reducing stress (Coyle et al. 2004; Husen Sharma 2014; Purbosari et al. 2019). One of the most used compounds is tricaine methanesulphonate (MS-222) (Chambel et al. 2015; Coyle et al. 2004; Topic Popovic et al. 2012). However, due to adverse effects (Felix et al. 2021, 2020; Misawa et al. 2014; Sladky et al. 1999; Wang et al. 2020), the focus has shifted toward the use of compounds of natural origin (Emeka et al. 2014; Medeiros Junior et al. 2018; Purbosari et al. 2019; Souza et al. 2019). Among the natural compounds, eugenol has been the most explored. It is a monoterpene compound and the major constituent of clove oil, and several biological properties have been demonstrated (Abbaszadeh et al. 2014; Khalil et al. 2017; Kheawfu et al. 2017; Ma et al. 2018; Pramod et al. 2010). A good anaesthetic capacity in different species of fish and different stages of development has also been reported (de Oliveira et al. 2019a, b; Ferreira et al. 2021; He et al. 2020; Medeiros Junior et al. 2018; Palić et al. 2006; Romaneli et al. 2018). However, developmental defects in zebrafish (Tao et al. 2023) and increased neuronal excitability in fish during short-term anaesthetic baths (Barbas et al. 2021) have been reported. Therefore, as evidence shows other monoterpenes are an attractive, effective alternative for fish anaesthesia (Félix et al. 2023), it is suggested to evaluate other compounds, such as thymol and menthol.

Thymol is a major compound of thyme oil exhibiting numerous properties, from antioxidant, antifungal, and antibacterial to sedative and anaesthetic properties in some aquatic species (Aydın & Orhan 2021; Souza et al. 2019; Yousefi et al. 2022, 2018). Menthol is found in plants such as mint and has numerous pharmacological properties of interest for aquaculture (Abbaszadeh et al. 2014; Bastaki et al. 2018; Dawood et al. 2020; Galeotti et al. 2002; Kamatou et al. 2013). It has also been successfully used as an analgesic and anaesthetic in several species of juvenile and adult fish (Ananias et al. 2022; Braz et al. 2017; de Padua et al. 2018; Façanha & Gomes 2005; Ferreira et al. 2021; Medeiros Junior et al. 2018; Pereira-da-Silva et al. 2016; Zapata‐Guerra et al. 2020). However, the information about its anaesthetic profile in the early stages of development is limited (Coyle et al. 2004; Rehman et al. 2017; Schroeder et al. 2021).

In aquaculture, animals are subjected to stressful situations from birth to adulthood (Rehman et al. 2017), and these situations can cause changes that alter the normal development of animals, thus damaging the entire production chain (Gabriel & Akinrotimi 2011; Husen & Sharma 2014; Martos-Sitcha et al. 2020; Souza et al. 2019). The zebrafish model is increasingly used as a universal model that represents the teleost family and is increasingly used in aquaculture in the most diverse areas, ranging from nutrition to immunological studies (Dahm & Geisler 2006; Jørgensen 2020; Lee‐Estevez et al. 2018; Ribas & Piferrer 2014; Ulloa et al. 2014). In particular, the initial phases of development are highly sensitive to compounds and external stimuli (Basnet et al. 2019; Rosa et al. 2022). Other advantages such as ease of maintenance, no legal restrictions up to 120 hpf (Strahle et al. 2012), similarity to and applicability in other species (Babin et al. 2014; Chang et al. 2022; Chowdhury et al. 2022; Garcia-Moreno et al. 2019; Massoz et al. 2021; Thienpont et al. 2011), the large number of replicates per assay, and the fact that by 72 hpf (hours post-fertilization), major neurotransmitters can be found (Felix et al. 2019; Rico et al. 2011) make it a useful model for anaesthesia research (Felix et al. 2019). In a previous work, it was possible to verify the similar anaesthetic profile of thymol and menthol to MS-222 and eugenol in zebrafish early life stages (Vieira, Sousa, et al. 2024). Based on the observed promising effects, the present work was planned to address the use of these compounds for transport conditions using the zebrafish early life stage model. The hypothesis is that the addition of these compounds to the transportation water will not significantly alter the analysed parameters and will produce outcomes comparable to those observed in the naïve (non-stressed) group, indicating their potential as alternatives for fish transportation.

## Materials and methods

### Chemicals

The ethyl 3-aminobenzoate methanesulfonate, 98% (MS-222, CAS: 886–86-2), was purchased from Merck (Algés, Portugal). Eugenol, 99% (CAS number: 97–53-0), and DL-menthol, 98% (CAS number: 89–78-1), were purchased from Alfa Aesar (Kandel, Germany) and thymol; extra pure (CAS number: 89–83-8) was purchased from EMD Millipore (Oeiras, Portugal). The protease inhibitor cocktail (CAS number: HY-K0010) was obtained from MedChemExpress (New Jersey, USA) and stored at − 20 °C. Sodium bicarbonate was used to neutralize the stock solution of MS-222 (1500 mg L^−1^) at a pH between 7.2 and 7.4. Stock solutions of eugenol (100 g L^−1^), thymol (35 g L^−1^), and menthol (89 g L^−1^) were prepared in a 1:10 water/ethanol mixture (Audira et al., 2020) and stabilized at a pH between 7.4 and 7.8. Solutions were prepared in autoclaved 1 × E3 medium (60 × stock: 5 mM NaCl, 0.17 mM KCl, 0.33 mM CaCl_2_, and 0.33 mM MgCl_2_, pH 7.2) (Meyers 2018). Until further dilution, all solutions were maintained at 4 °C.

### Zebrafish maintenance

The zebrafish AB and Tg(mpxGFP)^i114^ strains, the latter obtained from the European Zebrafish Resource Center (EZRC, Karlsruhe Institute of Technology, Eggenstein Leopoldshafen, Germany), were maintained under standard light (14:10 light:dark) and temperature (28 °C) conditions for the species and as previously described (Lanzarin et al. 2019; Vieira et al. 2021). Zebrafish reproduction involved pairing male and female zebrafish in a ratio of 2:1, in small boxes overnight. The fertilized eggs were collected and bleached with a Chloramine-T solution (0.5% w/v) (Teixido et al. 2019). The eggs were then rinsed twice with E3, selected under a stereomicroscope (Varga 2011; Westerfield 2007), allowed to develop, and used according to the subsequent experiments. The laboratory experiments that involved animals were conducted in strict accordance with ethical principles and the EU directive (2010/63/EU) as well as national regulations for animal experimentation and welfare (particularly, Decreto-Lei 113/2013).

### 4 h-LC_50_ determination

To confirm the safety of the compounds, the OECD testing guideline 236 was implemented to ascertain the lethal concentration that results in 50% mortality (LC_50_). Twenty 96 h post fertilization (hpf) larvae were arbitrarily distributed in 6-well plates (5 mL solution) for triplicate exposure to test solutions for a period of 4 h. This period of exposure corresponds to a short-term transport (Félix et al. 2021; Gebresenbet et al. 2011; Rebouças et al. 2019;  Sampaio and Freire 2016; Talling et al. 1998) and allowed the calculation of the lethal concentration. By doing this, it was guaranteed that the concentrations used were safe for the animals during the 4 h of transport simulation (Spanghero et al. 2019). E3 medium was employed as a blank control and to produce all test solutions of menthol (1.0, 7.5, 15, 25, 50, 100, 150, 200, 300, 600, 650, 700, and 1000 mg L^−1^) and thymol (1.0, 7.5, 15, 20, 50, 60, 65, 70, 75, 80, 85, 90, 100, 200, and 300 mg L^−1^). After the exposure, probit analysis was used to determine the 4-h LC_50_ values (Finney 1971). The 4-h LC_50_ and the 95% confidence limits were calculated as 182.3 (174.9–189.9) mg L^−1^ for menthol and 71.32 (70.72–71.92) mg L^−1^ for thymol (Figure S1). Based on the calculated LC_50_, one sub-lethal concentration that caused no mortality during the exposure window was selected for each compound. The selected concentrations were 15 mg L^−1^ for thymol and 50 mg L^−1^ for menthol.

### Transport simulation event

To evaluate the suitability of these compounds for a transport situation, zebrafish were transported for 4 h to simulate the typical duration of brief and regional transportation (range 4 to 8 h) reviewed in F. D. F Sampaio and C. A. Freire (2016). Ninety-six hpf larvae were used according to previous work (Castillo-Ramírez et al. 2019; Faught & Vijayan 2018). Thirty zebrafish larvae with 72 hpf (3 dpf) were transferred to quintuple 50 mL centrifugal tubes on the evening prior to the experiment. The tubes contained a final volume of 20 mL of E3 medium and each of the following six experimental conditions: a naïve group without transport simulation, a control group with transport simulation, and anesthetized groups with MS-222 (MS-222, 200 mg L^−1^), the eugenol (Eu80, 80 mg L^−1^), the thymol (T15, 15 mg L^−1^), and menthol (M50, 50 mg L^−1^), which were also transport-simulated. The transportation event simulation was carried out by placing the tubes containing the larvae on a shaker and subjecting them to vortex stimulation (200 rpm) at a mean acceleration of 4.8 ± 0.3 m s^−2^ and a noise of 76.9 ± 1.1 dB, mimicking the conditions observed in the transport of live animals (Félix et al. 2021; Gebresenbet et al. 2011; Rebouças et al. 2019; Talling et al. 1998).

### Physiological markers

#### Heartbeat rate

By the end of the transportation event, the heart rate was analysed in 10 animals per group, briefly, random larvae were collected from each group and analysed under a SMZ800 stereomicroscope for 30 s each and then converted to heart rate per minute. The naïve and the control group were immobilized in 3% methylcellulose before recording the heart rate (Muntean et al. 2010).

#### Metabolic alterations

##### Metabolic rate

The metabolic rate is an important parameter providing insights into the stress and anaesthetic effects, energy expenditure, homeostasis, osmoregulation, and oxygen consumption (Biswal et al. 2021; Sopinka et al. 2016; Treberg et al. 2016). The metabolic analysis was adapted from the Redi et al. (2018) protocol. The preparation of resazurin, the number of larvae used, and the proceedure of the assay and reading of the results were carried out according to the procedure adapted and optimized previously (Vieira et al. 2025).

##### Enzymatic activity (ATPase and LDH)

To evaluate the potential effects of larvae transportation under thymol and menthol sedation, relevant enzymatic markers were analysed (de Oliveira, Lemos et al., 2019a; Félix et al. 2021; Mirzargar et al. 2022; Wang et al. 2023). Five new replicate exposures (30 larvae per replicate, five replicates per concentration) were performed as described earlier. Following the transportation event, the larvae were rinsed three times with E3 medium before being collected and homogenised in cold buffer (Deng et al. 2009) using a Tissuelyser II (30 Hz for 30 s; Qiagen, Hilden, Germany). The supernatant was used to assess the total ATPase activity by measuring inorganic phosphate (Pi) released using ammonium molybdate at 820 nm (Lanca et al. 2015) and to assess the lactate dehydrogenase (LDH) activity by the oxidation of NADH at 340 nm (Domingues et al. 2010). The protein level of the samples (Noble and Bailey 2009) was used to normalize the data.

#### Stress parameters (cortisol, glucose and lactate)

In order to evaluate how larvae respond to the stress caused by the transportation event, the most used markers, namely, cortisol, glucose, and lactate, were assessed (Lemos et al. 2023; Sampaio and Freire 2016; Vaage et al. 2023). Five new replicates of 30 larvae were collected immediately following the stress-inducing event for cortisol quantification. Animals were promptly euthanised with an overdose of MS-222 (0.4 g L^−1^) and transferred to microtubes containing 100 µL of PBS and a protease inhibitor cocktail (1: 100, v:v). The protease inhibitor cocktail was added to prevent the rapid degradation of samples by protease enzymes. To assess cortisol levels, samples were treated as described before (Vieira, Venâncio, et al. 2024; Vieira et al. 2025). Cortisol quantification proceeded using a Cortisol Enzyme Immunoassay kit (Arbor Assaystm DectectX®, Ann Arbor, USA). New samples were collected for glucose and lactate quantification, homogenised as before, and centrifuged at 12,000 × g for 10 min at 4 °C (Brun et al. 2019). The used kits for glucose were ref. 1,001,200, Spinreact, Barcelona, Spain, and for lactate ref. 1001330, Spinreact, Barcelona, Spain.

### Biological markers

#### Oxidative stress

Total reactive oxygen species (ROS), enzymatic activities of superoxide dismutase (SOD), catalase (CAT), glutathione peroxidase (GPx), glutathione reductase (GR), and glutathione-s-transferase (GST), and levels of reduced glutathione (GSH), oxidized glutathione (GSSG), oxidative stress index (ISO), protein carbonylation (PC), lipid peroxidation (LPO), and nitric oxide (NO) were evaluated using the same supernatant obtained in “Enzymatic activity (ATPase and LDH).” The ROS were determined using the fluorescent probe DCFH-DA at 485 nm and 530 nm corresponding to the excitation and emission wavelengths, respectively (Deng et al. 2009). The SOD activity was assayed by its ability to inhibit the photochemical reduction of nitrobluetetrazolium (NBT) at a wavelength of 560 nm (Durak et al. 1993), while CAT activity was detected by monitoring the catalytic decomposition of hydrogen peroxide at 240 nm (Claiborne 1985). The GPx and GR activities were measured by monitoring the oxidation and subsequent reduction of NADPH at 340 nm (Massarsky et al. 2017). The GST activity was evaluated according to Habig and Jakoby (1981) method, through the conjugation of 1-chloro-2,4-dinitrobenzene (CDNB) with GSH at 340 nm. The GSH and GSSG levels were quantified after derivatization with ortho-phthalaldehyde (OPA) and measured at 320 nm and 420 nm for excitation and emission wavelengths, respectively (Gartaganis et al. 2007). The OSI was calculated as the ratio GSH: GSSG. The PC levels were determined using the DNPH extinction coefficient at 450 nm (Mesquita et al. 2014). The LPO level was assayed quantifying thiobarbituric acid reactive substances (TBARS), following the method described by Wallin et al. (1993) at 530 nm for MDA-TBA adducts and at 600 nm for non-specific reactive substances.

#### NRF2 levels

The Western blot technique was used to quantify the expression of specific proteins involved in oxidative stress and cellular death pathways (Fan et al. 2021; Yu et al. 2019; Zhao et al. 2015). New replicates were collected (*n* = 5), under the conditions described above, with larvae collected at 96 hpf and processed as reported before (Félix 2024). Collected larvae were washed in PBS and lysed in RIPA buffer (50 μL) utilising a cordless motor and pellet mixer (VWR International, Portugal). The resulting homogenates were centrifuged, supernatants collected, and protein content determined by the BCA method (Walker 2009). The samples, containing approximately 30 μg of protein, were separated on a 12% sodium dodecyl sulphate (SDS)-polyacrylamide gel at 120 V and 4 °C in running buffer (192 mM glycine, 1% SDS, and 25 mM Tris-Base, pH 8.3). The transfer to PVDF membrane was conducted as described before (Félix 2024). The primary antibodies for GAPDH (1:1000, GTX100118, 36 kDa, GeneTex) and Nrf2 (1:20, PCRP-NFE2L2–1D12, 68 kDa, DSHB) were used overnight following the blockade of the membrane with 5% BSA. The secondary antibodies used was 1:5000, mouse IgGκ BP-HRP, sc-516102-CM, and rabbit IgG HRP, sc-2357 from Santa Cruz Biotechnology, USA. Utilising the ImageJ software, the TMB stained bands were quantified (Gallo-Oller et al. 2018) and the resulting values normalized to the control levels.

#### Apoptosis and DNA damage

To evaluate Caspase 3 and 9 levels, the supernatant collected in “Enzymatic activity (ATPase and LDH)” was used. Caspase 3 and 9 levels were quantified by measuring pNA at 405 nm from specific substrates (Ac-DEVD-pNA and Ac-LEHD-pNA, respectively, for caspase 3 and 9) (Mincberg et al. 2011). Caspase 3 and AIF levels were also evaluated by the western blot technique as described in the final of section 2.6.2. The primary antibodies for these assessments were anti-AIF (1:1000, GTX113306, 67 kDA, GeneTex) and anti-casp 3 (1:5000, GTX300110, 32 kDA, GeneTex). The DNA damage was quantified based on an adaptation of the alkaline precipitation assay described by Olive (1988). For this, five new replicate exposures (30 larvae per replicate, five replicates per concentration) were performed, washed, and homogenised as before. Tissue homogenate (25 μL) was mixed with 200 μL of 2% SDS containing 10 mM EDTA, 10 mM Tris-base, and 40 mM NaOH and vortexed for 1 min. Alkaline precipitation occurred when 200 μL of 0.12 M KCl was added. This mixture was further heated at 60 °C for 10 min, mixed again and cooled at 4 °C for 30 min. After this step, the samples were centrifuged at 8000 g for 5 min at 4 °C. To 50 μL of sample, 150 μL of Hoechst dye (1 μg mL^−1^, in buffer containing 0.4 M NaCl, 4 mM sodium cholate, and 0.1 M Tris‐acetate) was added. The fluorescence was then measured at 360 nm excitation and 450 nm emission wavelengths in Cary Eclipse fluorescence spectrophotometer (Varian, Palo Alto, USA). To detect whether compounds trigger the apoptotic pathway, which may relate to oxidative stress and other pathways (Félix et al. 2020; Ge et al. 2023), the acridine orange (AO) staining was used according to the methodology previously described (Felix et al. 2014). (Félix et al. 2020; Ge et al. 2023). After the transportation event, larvae were incubated for 30 min in the dark, in a solution of 5 µg mL^−1^ of acridine orange (AO) at room temperature (Felix et al. 2014; Lanzarin et al. 2021). After, larvae were washed 3 × with E3 solution to remove the excess of AO, animals were observed within an inverted microscope (IX 51, Olympus, Antwerp, Belgium) using a Fluorescein-Isothiocyanate (FITC) filter. The total fluorescence was quantified using the ImageJ program (National Institutes of Health of the USA, Bethesda, MD, USA).

#### Inflammatory evaluation

The Tg(mpxGFP)^i114^, which specifically features neutrophils with green fluorescent protein, was used to quantify and observe inflammatory processes (Elks et al. 2011; Renshaw et al. 2006). Transgenic embryos were obtained in the same way as the AB line and, following the transportation event, fluorescent images were obtained as described for AO staining. Neutrophil number was quantified in the total area of all animals manually using the ImageJ2 program (version 2.0.0, National Institutes of Health of the USA, Bethesda, MD, USA) (Grishagin 2015; Lanzarin et al. 2023). NO levels were determined using an adaptation of the Griess method at 540 nm (Krishnan and Kang 2019). Data was normalized by the protein content (Noble and Bailey 2009) after measurement in a PowerWave XS2 microplate scanning spectrophotometer (Bio-Tek Instruments, USA).

### Data analysis

The data normality was assessed using the Shapiro–Wilk normality test, and the variance homogeneity was assessed using the Brown-Forsythe test before the ANOVA. The Dunnett’s comparison test was employed to analyse the differences between groups and control when the data had a normal distribution. The data was reported as mean ± standard deviation. Despite the violation of the homogeneity of variances assumption in some parameters, ANOVA is generally robust to moderate violations. In these cases, the data followed a normal distribution, the group sizes were similar, but the homogeneity of variances was not met. Given visible differences in the graphs and the higher statistical power of parametric tests, ANOVA was chosen to detect group differences even when homogeneity of variances was not met. Also, the Student’s *t* test was used to compare the naïve group and the control group in all parameters. The statistical analysis was conducted using the GraphPad Prism software (version 9), with a significance level of *p* < 0.05.

## Results

### Physiological stress markers

**Fig. 1:**
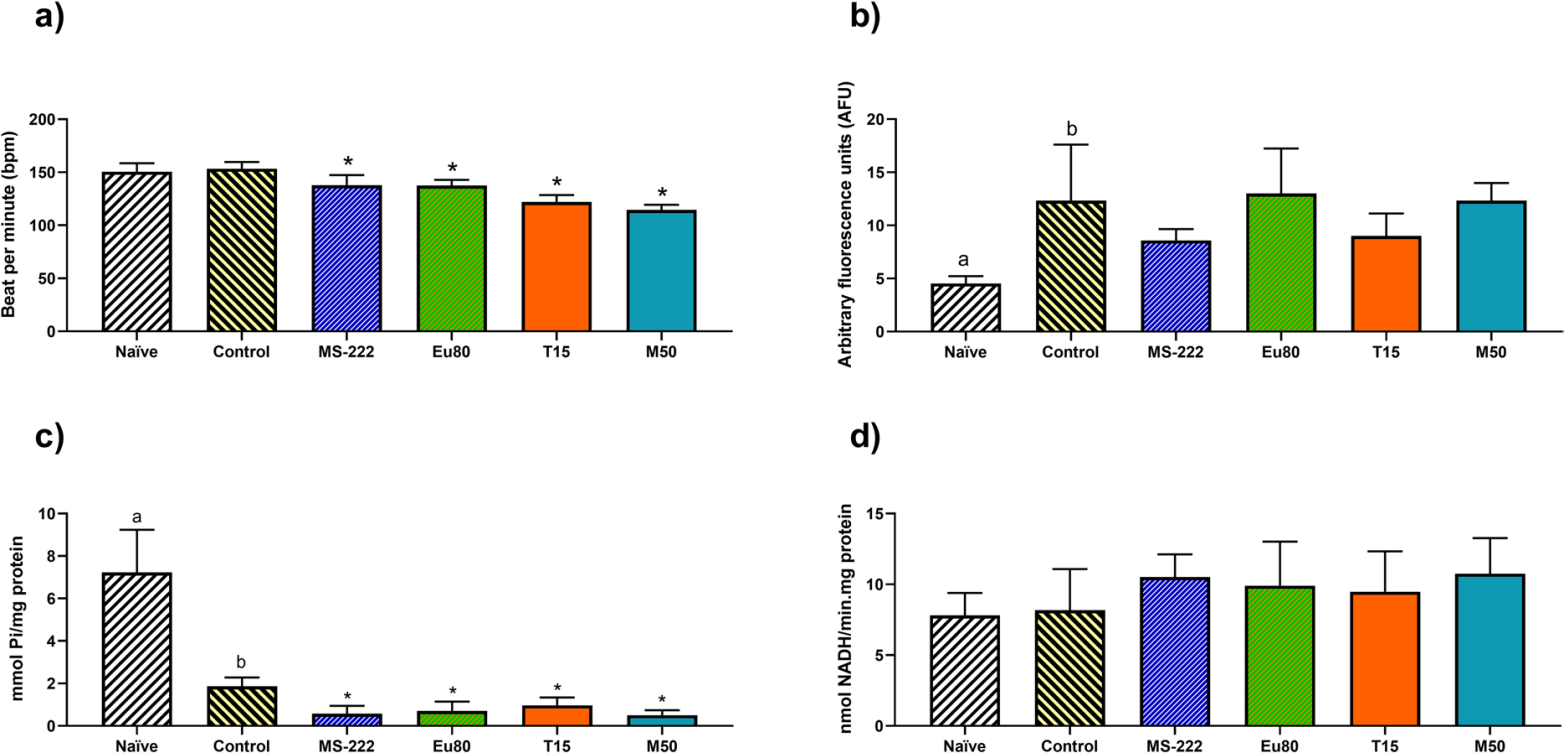
**a** Heartbeat rate, **b** metabolic rate, **c** ATPase activity and **d** LDH activity in 96 hpf zebrafish larvae. Data are expressed as mean ± SD for parametric data distribution. Statistical analysis was performed using Student’s *t* test (unpaired) to compare the naïve group with the control group, and used one-way ANOVA followed by Dunnett’s multiple-comparison test for comparation of the control and anaesthetized groups. Different lowercase letters indicate significant differences between the naïve and control groups (*p* < 0.05), and asterisks indicate significative differences between the control and anaesthetized groups (*p* < 0.05)

**Fig. 2:**
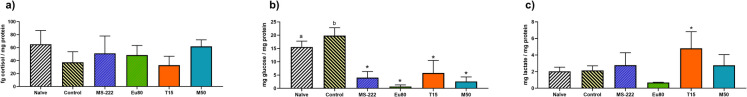
**a** Cortisol levels, **b** glucose levels, and **c** lactate levels in 96 hpf zebrafish larvae. Data are expressed as mean ± SD for parametric data distribution. Statistical analysis was performed using Student’s *t* test (unpaired) to compare the naïve group with the control group, and used one-way ANOVA followed by Dunnett’s multiple-comparison test for comparation of the control and anaesthetized groups. Different lowercase letters indicate significant differences between the naïve and control groups (*p* < 0.05), and asterisks indicate significative differences between the control and anaesthetized groups (*p* < 0.05)

The results of the physiological measurements are shown in Figs. 1 and 2, while the statistical outcomes are presented in table S1. Following the 4-h transportation event, there was no difference between the control and naïve group (*p* = 0.5565) relative to the heart rate. Yet, the animals that were anesthetized and transported for 4 h showed a significant decrease in their heart rate (Fig. 1a) when compared to the control and naïve groups (*p* < 0.0001). The metabolic rate (Fig. 1b) increased significantly in the control transported animals compared to the naive group (*p* = 0.0111). In anesthetized animals subjected to the transportation event, no significant differences were observed compared to the control group (*p* = 0.130); however, their metabolic rate was significantly higher than that of the naive group (*p* < 0.0001). The analysis of the enzymatic activity revealed that transportation significantly reduced ATPase activity (Fig. 1c) compared to the naive group (*p* = 0.0004). The use of anaesthetics resulted in an even greater reduction in ATPase activity compared to the control group (*p* < 0.0001). No significant differences in LDH activity (Fig. 1d) were observed in animals subjected to anaesthesia and transportation. Similarly, no statistical differences were observed in cortisol levels (Fig. 2a) following transportation under anaesthesia. However, glucose and lactate levels were significantly affected (Fig. 2 b and c, respectively). Transportation caused an increase in glucose levels compared to the naive group (*p* = 0.033), which was reduced using anaesthesia (*p* < 0.0001), bringing glucose levels to values even lower than those observed in the naive group (*p* < 0.001). Transportation had no significant effect on lactate levels; however, an increase was observed when thymol was used (*p* = 0.015 and *p* = 0.020 for the naïve and control group, respectively).

### Oxidative-stress related markers

**Fig. 3:**
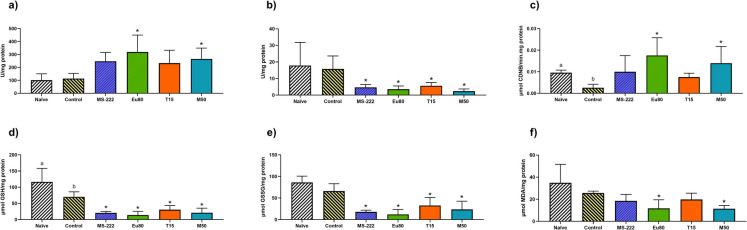
**a** SOD; **b** CAT; **c** GST; **d** GSH; **e** GSSG; **f** LPO in 96 hpf zebrafish larvae. Data are expressed as mean ± SD for parametric data distribution. Statistical analysis was performed using Student’s *t* test (unpaired) to compare the naïve group with the control group, and used one-way ANOVA followed by Dunnett’s multiple-comparison test for comparation of the control and anaesthetized groups. Different lowercase letters indicate significant differences between the naïve and control groups (*p* < 0.05), and asterisks indicate significative differences between the control and anaesthetized groups (*p* < 0.05)

The significant response of different biochemical markers following the transportation is presented in Fig. 3, while statistical data is shown in Table S2. Overall, no significant changes in ROS production were observed during transportation or with the use of anaesthesia. Yet, the analysis of the different oxidative stress-related biomarkers revealed significant differences between treatments. While no differences were observed between the control and naive groups, all anesthetized groups showed an increase in SOD activity (Fig. 3a). This increase was statistically significant for eugenol and menthol compared to both the naive (*p* < 0.01) and control (*p* < 0.05) groups. An opposite trend was observed for CAT activity (Fig. 3b), with all anesthetized groups showing a significant decrease in activity compared to both control groups (*p* < 0.05). No significant differences were observed between the naive and control groups. For the GST activity (Fig. 3c), transportation caused a significant reduction compared to the naive group (*p* = 0.0002). However, when anaesthesia was used during transportation, the GST activity increased to levels comparable to the naive group (*p* > 0.05). Additionally, eugenol and menthol significantly elevated GST activity compared to the control group (*p* = 0.004 and *p* = 0.029, respectively). The transportation also caused a significant reduction of GSH levels (Fig. 3d; *p* = 0.048), which was not observed for the GSSG levels (Fig. 3e). Nonetheless, the use of anaesthesia during larvae transportation led to a significant reduction in both GSH and GSSG levels compared to the naive and control groups (*p* < 0.01). Despite these variations, the OSI remained unaffected. The MDA levels (Fig. 3f) were not affected by transportation; however, the use of eugenol and menthol significantly reduced MDA levels compared to both the control and naive groups (*p* < 0.01). No changes were observed for the GPx and GR activity nor for the levels of protein carbonylation.

### Nrf2 levels

**Fig. 4:**
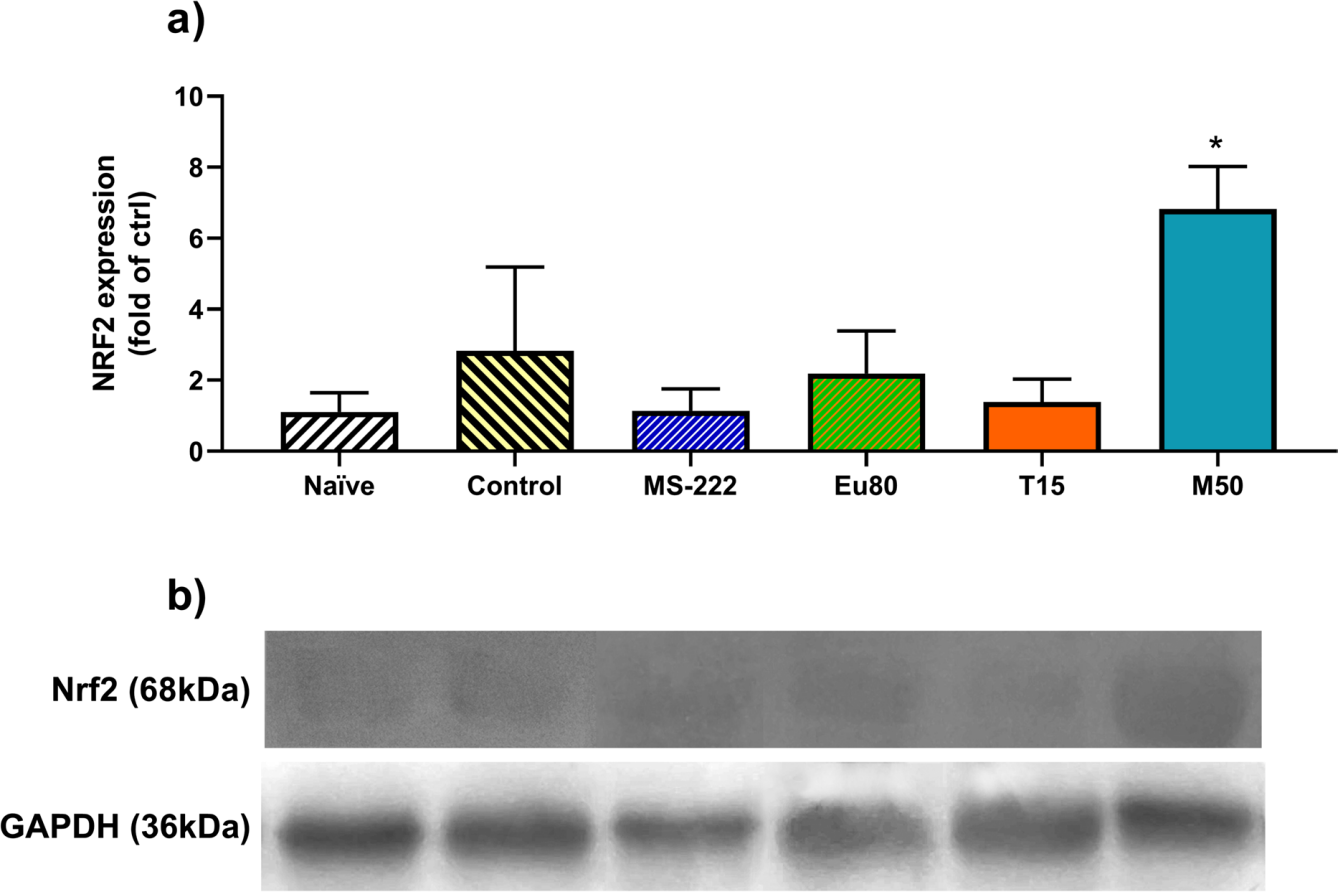
**a** Nrf2 protein levels in 96 hpf zebrafish larvae following 4 h transportation. **b** Representative Western blots of Nrf2. Data are expressed as mean ± SD for parametric data distribution. Statistical analysis was performed using Student’s *t* test (unpaired) to compare the naïve group with the control group, and used one-way ANOVA followed by Dunnett’s multiple-comparison test for comparation of the control and anaesthetized groups. Asterisks indicate significative differences between the control and anaesthetized groups (*p* < 0.05)

The levels of the Nrf2 factor were determined by western blot and the results obtained are presented in Fig. 4, while the original data are shown in Table S2. The transportation caused a slight increase in Nrf2 expression levels, and variations were observed for the anaesthetized transported groups. Yet, a significant increase was observed following transportation under menthol anaesthesia with a sixfold (*p* < 0.0001) and twofold (*p* = 0.001) increase in its levels compared to the naïve and control groups, respectively.

### Apoptosis and DNA damage

**Fig. 5:**
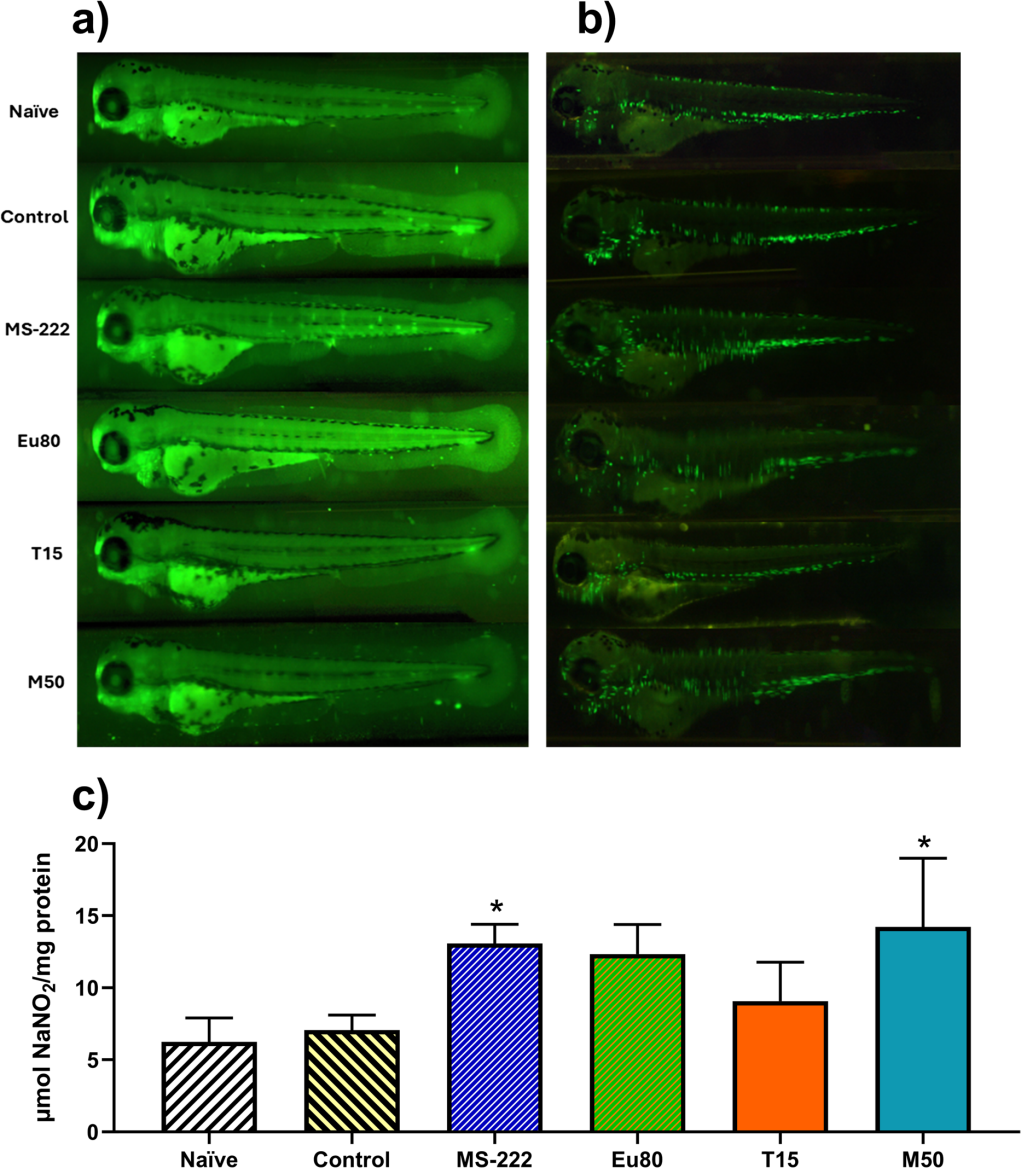
Effect of different anaesthetics on apoptosis and inflammation in 96 hpf zebrafish larvae. **a** Illustrative images from larvae exposed to the AO probe. **b** Illustrative images from Tg(mpxGFP)i114 larvae used in the assay. **c** Graphic of NO levels. The scale bar represents 500 µm. Data are expressed as mean ± SD for parametric data distribution. Statistical analysis was performed using Student’s *t* test (unpaired) to compare the naïve group with the control group, and used one-way ANOVA followed by Dunnett’s multiple-comparison test for comparation of the control and anaesthetized groups. Asterisks indicate significative differences between the control and anaesthetized groups (*p* < 0.05)

The data regarding the different apoptosis-related markers and DNA damage are shown in Table S2. Overall, no significant changes were observed for the apoptosis signals (Fig. 5a), or for the DNA damage.

### Inflammatory analysis

Regarding the inflammatory analysis determined using the Tg(mpxGFP)^i114^ (Fig. 5b), no significant differences were observed between transported animals and the naïve group. When assessing NO levels (Fig. 5c), an indicator of inflammation, no differences were observed between the control transported group and the naive group. However, the use of anaesthesia increased NO levels, with MS-222 (*p* = 0.031) and menthol (*p* = 0.007) showing significant differences compared to the control group. Compared to the naive group, MS-222, eugenol, and menthol all resulted in significant increases in NO levels (*p* < 0.05).

## Discussion

Aquaculture practices subject animals to various stressors that can disrupt normal development and impact the production chain (Ashley 2007; Barreto et al. 2021; Mancuso 2013). Zebrafish serve as a widely recognized model for studying teleost fish and are frequently used in aquaculture research (Dahm and Geisler 2006; Jørgensen 2020; Lee‐Estevez et al. 2018; Ribas and Piferrer 2014; Ulloa et al. 2014). This study builds on the previous anaesthetic properties of thymol and menthol in early life stages of zebrafish (Vieira, Sousa, et al. 2024) and was planned to evaluate the efficacy of these compounds during a simulated fish transport (4 h), hypothesizing that their inclusion in transport water would not significantly affect physiological, oxidative stress, or inflammatory markers while providing stress-reducing benefits. The results show that transport increases metabolic rates and reduces ATPase activity compared to the naïve group, while the use of anaesthetics mitigated glucose spikes caused by transportation and reduced ATPase activity and the heart rate. Regarding oxidative-stress-related markers, GST activity, reduced during transportation, was restored by anaesthetics to naïve levels. Transportation also caused a reduction in GSH levels, which were further reduced by anaesthetics; a phenomenon also observed in GSSG levels, although the oxidative stress index (OSI) was not affected. Transportation under anaesthesia also induced an elevation of SOD levels while reducing CAT activity and MDA levels. Transportation slightly increased Nrf2 levels, with menthol anaesthesia causing a significant six-fold increase compared to naïve groups. No significant changes were observed in apoptosis and DNA damage markers. While inflammatory NO levels increased with anaesthetics, no overt inflammatory responses were observed.

In this study, a vibrational platform was employed to generate a water vortex flow, effectively simulating transport-related stress in zebrafish (Castillo-Ramírez et al. 2019; Fang et al. 2023; Fuzzen et al. 2010). This method mimics the physical and environmental stimuli associated with transportation (Fang et al. 2023; Vieira et al. 2025) and elicits physiological responses, such as an elevated cortisol level, comparable to those observed in zebrafish exposed to other stressors (Barcellos et al. 2007; Castillo-Ramírez et al. 2019; Eto et al. 2014; Mikloska et al. 2022; Pavlidis et al. 2013; Yeh et al. 2013). In this setup, zebrafish larvae exhibit a natural tendency to orient themselves against the current created by the vortex, a behaviour that demands significant energy expenditure (Castillo-Ramirez et al. 2024; Castillo-Ramírez et al. 2019). This elevated activity triggers a cortisol response through the activation of the hypothalamus-pituitary-interrenal (HPI) axis, a central component of the stress response system (Schreck and Tort 2016). However, following the transportation simulation, cortisol levels were found unchanged. This lack of differences in cortisol levels can be attributed to the negative feedback mechanism of the HPI axis. Following an initial cortisol peak induced by stress, this mechanism effectively regulates cortisol levels, gradually reducing them as the organism habituates to the stressor. Supporting this observation, previous studies have shown that cortisol levels typically peak at the onset of a stress event, but rapidly decrease within a short time, returning to baseline levels (Castillo-Ramirez et al. 2024; Fuzzen et al. 2010; Ramsay et al. 2009). While the observed normalization of cortisol levels post-transportation reflects the efficacy of the HPI axis’s negative feedback mechanism in responding to acute stress, it does not preclude the possibility of chronic stress. The referred studies focus on acute exposure to stressors, contrary to the present study, where 4 h exposure to a stressful stimulus was applied. Chronic stress arises from prolonged or repeated exposure to stressors (Balasch and Tort 2019; Schreck and Tort 2016) and may manifest as dysregulation of the HPI axis (Best et al. 2023; Chin et al. 2022; Virtanen et al. 2023), leading to sustained physiological changes that are not immediately apparent through cortisol measurements. Persistent stress, such as continuous exposure to vortex-induced currents, can result in other subtle but significant alterations in zebrafish physiology or behaviour. Supporting this idea, studies in fish have shown that chronic stress can lead to impacts on different physiological parameters even in the absence of elevated cortisol levels (Ellis et al. 2012; Fast et al. 2008; Laberge et al. 2019). Investigating these could provide insight into whether zebrafish are experiencing chronic stress despite the apparent normalization of cortisol. Despite this, and given the quick normalization of cortisol, no significant differences in cortisol levels were anticipated with the use of anaesthetics, as the stress-induced hormonal fluctuations would have already stabilized. Therefore, while further time-dependent studies are needed to clarify this, the anaesthetics had a more pronounced effect on other physiological aspects, such as the heart rate. This is consistent with the sedative effects of anaesthetics on the circulatory system (Aydın, 2020; Aydın and Barbas 2020; Leyden et al. 2022; Reid et al. 2022; Yousaf et al. 2022). Additionally, transport caused an elevation of the metabolic rate, glucose levels, and a decrease in ATPase activity. While cortisol is often the primary marker of HPI activation, other physiological processes can be affected by stress without significant changes in cortisol levels (Lemos et al. 2023; Portz et al. 2006; Thau et al. 2024), with these variations suggesting a complex physiological response to stress. For instance, the activation of the HPI can lead to an initial increase in the metabolic rate and glucose levels as a part of the adaptive response to the stressor (Balasch and Tort 2019; Barton 1988; Santos et al. 2010; van der Kooij 2020), thereby ensuring that there are enough energy resources to cope with the challenge (Martínez-Porchas et al. 2009; Santos et al. 2010; Sopinka et al. 2016). An increase in glucose levels was indeed observed, possibly stimulated by an initial peak in cortisol, which promotes gluconeogenesis (Barton 1988; Fang et al. 2023). Alternatively, the glucose increase could result from stress-induced activation of catecholamine-mediated pathways (Fabbri et al. 1998; Gesto et al. 2014; Martínez-Porchas et al. 2009; Portz et al. 2006; Sampaio and Freire 2016). Still, the anaesthetic’s effects were sufficient to mitigate the stress-induced glucose response, as evidenced by glucose levels being reduced even further than those observed in the naïve group. This reduction in glucose levels suggests that the anaesthetics may have induced or supported a hypometabolic state. In this state, metabolic demands are reduced, potentially mirroring the physiological strategy employed by some species under severe hypoxia or anoxia (Zhang et al. 2021), where they temporarily downregulate energy-intensive processes to conserve energy and lower basal oxygen requirements. The observed decrease in ATPase activity induced by transportation, as previously reported (Vieira et al. 2025), and its further suppression by anaesthetics, alongside a reduction in heart rate, strongly corroborate this hypometabolic state as a protection mechanism to conserve energy and prevent cellular damage (Jiang et al. 2023; Kadamani et al. 2024; Welker et al. 2013). It is worth noting that the prolonged exposure to thymol anaesthesia may have exacerbated these effects, as extended metabolic suppression can further shift energy production towards anaerobic pathways, resulting in an increased production of lactate as a byproduct of anaerobic glycolysis in juvenile fish (Yousefi et al. 2022) and other animals (Bao et al. 2011). Notwithstanding, based on the observed patterns and the critical role of glucose in metabolic pathways during early developmental stages (Thompson et al. 2024), further studies should focus on unravelling the mechanisms underlying these responses, which can be a consequence of the HPI activation even in the absence of sustained cortisol elevation.

Albeit this, the increase in glucose observed after transportation can be associated with a compromise in the organism’s antioxidant capacity (González et al. 2023), triggering a shift in metabolic pathways to cope with the stress-induced cellular damage. In this context, the literature suggests that high glucose concentrations induce changes in the redox balance, leading to alteration of the redox regulatory capacity of Nrf2 (Albert-Garay et al. 2022), a key protein involved in managing oxidative stress (Ma 2013). This alteration is thought to be mediated by NF-kB, a transcription factor that is often activated during inflammation or stress (Albert-Garay et al. 2022; Ma 2013). However, the results observed after 4 h of transportation in zebrafish larvae did not align with these expectations. Despite a decrease in GST activity, similar to what was observed before (Vieira et al. 2025), and a decrease in GSH levels, which could be indicative of oxidative stress, no changes in Nrf2 expression or other biomarkers of inflammation or stress were observed in the control group after transportation, as observed previously (Mirzargar et al. 2021; Ren et al. 2022). Overall, the lack of changes in these biomarkers could be explained by the negative feedback regulation of the HPI axis described before. Once cortisol levels peaked and initiated the necessary adaptive responses, the body likely returned to baseline, reducing inflammation and stress marker expression. This mechanism has been documented in various studies (Chatzopoulou et al. 2016; Kalamarz-Kubiak 2018; Tort 2011; van den Bos et al. 2020), highlighting the importance of further research to confirm this hypothesis. Despite this, when examining the impacts of transportation under different anaesthetic compounds, variations in Nrf2 levels were observed alongside an increase in NO levels, suggesting that transportation under anaesthesia induced a stress response involving both antioxidant and inflammatory pathways. In this context, anaesthetics are believed to impair various aspects of the inflammatory response, either by indirectly altering the stress response or by directly interfering with immune cell function (Cruz et al. 2017), although there is a lack of information regarding these effects of anaesthetics in fish specifically. Notwithstanding, menthol exposure resulted in a significant increase of Nrf2 levels, consistent with previous observations (Carneiro et al. 2022), highlighting its potential role in modulating oxidative stress responses during transport. However, there remains a notable lack of information regarding the specific effects of these compounds on these pathways. Additionally, the increased SOD activity and decreased CAT activity suggest, respectively, an initial attempt to neutralize superoxide radicals while the ability to handle hydrogen peroxide, the downstream product of SOD, has been impaired. This imbalance could reflect a shift in the overall antioxidant defence system, a phenomenon that has already been documented following exposure to these types of compounds (Carneiro et al. 2022; Krishnan et al. 2019; Vieira, Sousa, et al. 2024). It is possibly due to limitations in enzymatic activity, a depletion of cellular resources or redirection of metabolic pathways by the anaesthetics under prolonged stress that needs further investigation. On the other hand, the GST activity was restored to control values, which could indicate a recovery or compensation in the detoxification processes (Tierbach et al. 2018). However, the further decrease in GSH levels compared to the naive and control groups raises concerns as GSH is a crucial antioxidant that directly participates in neutralizing ROS and maintaining cellular redox balance (Georgiou-Siafis & Tsiftsoglou 2023). This depletion could reflect a limitation in the glutathione-dependent antioxidant system under prolonged anaesthetic exposure, which could potentially lead to cellular damage (Liu et al. 2022; Mytilineou et al. 2002). However, no such damage was observed, as evidenced by the lack of changes in cellular death-related parameters. Interestingly, lipid peroxidation levels were decreased in the anaesthetic-treated groups, suggesting that, despite the altered antioxidant enzyme activity and decreased GSH levels, the overall impact on cellular membranes was less severe than expected. Overall, these findings suggest a complex, paradoxical interplay between different pathways that may reflect an adaptive response to the anaesthetics and require further clarification.

In summary, the findings suggest that by the end of 4 h on the vibrating platform, cortisol levels were at the baseline levels due to the normal negative feedback mechanism that occurs in a stress response under control of the HPI axis. Changes in metabolic rate, glucose levels, and ATPase activity indicated a complex transportation-induced stress response. Anaesthetics, particularly thymol, lowered glucose levels, suggesting a shift to a hypometabolic state. Although oxidative stress markers like GST and GSH were altered, no changes in Nrf2 or inflammation biomarkers were observed, likely due to HPI regulation. However, anaesthetics, especially menthol, seemed to modulate oxidative stress through Nrf2 activation, warranting further investigation. Despite these changes, lipid peroxidation was reduced, suggesting less cellular damage. Overall, while more research is needed to understand the interactions between stress responses and anaesthetic effects, the results suggest that anaesthetics such as MS-222, eugenol, thymol, and menthol do not significantly disrupt physiological responses during early development under stress. This supports their potential use in fish transport during early stages and suggests that thymol and menthol could be viable alternatives that help avoid some of the negative effects associated with traditional anaesthetics, supporting their potential use for early developmental fish transportation.

## Supplementary Information

Below is the link to the electronic supplementary material.

ESM 1 (PNG 120 KB)

High Resolution Image (TIF 604 KB)

Supplementary file2 (DOCX 32 KB)

## Data Availability

No datasets were generated or analysed during the current study.
