# Supplementary figures and images for: Thymol and menthol as anaesthetics for short transportation of zebrafish larva
Source: Fish Physiol Biochem. 2025 Jul 30;51(4):129. doi: 10.1007/s10695-025-01530-x (PMC12310772; doi:10.1007/s10695-025-01530-x)

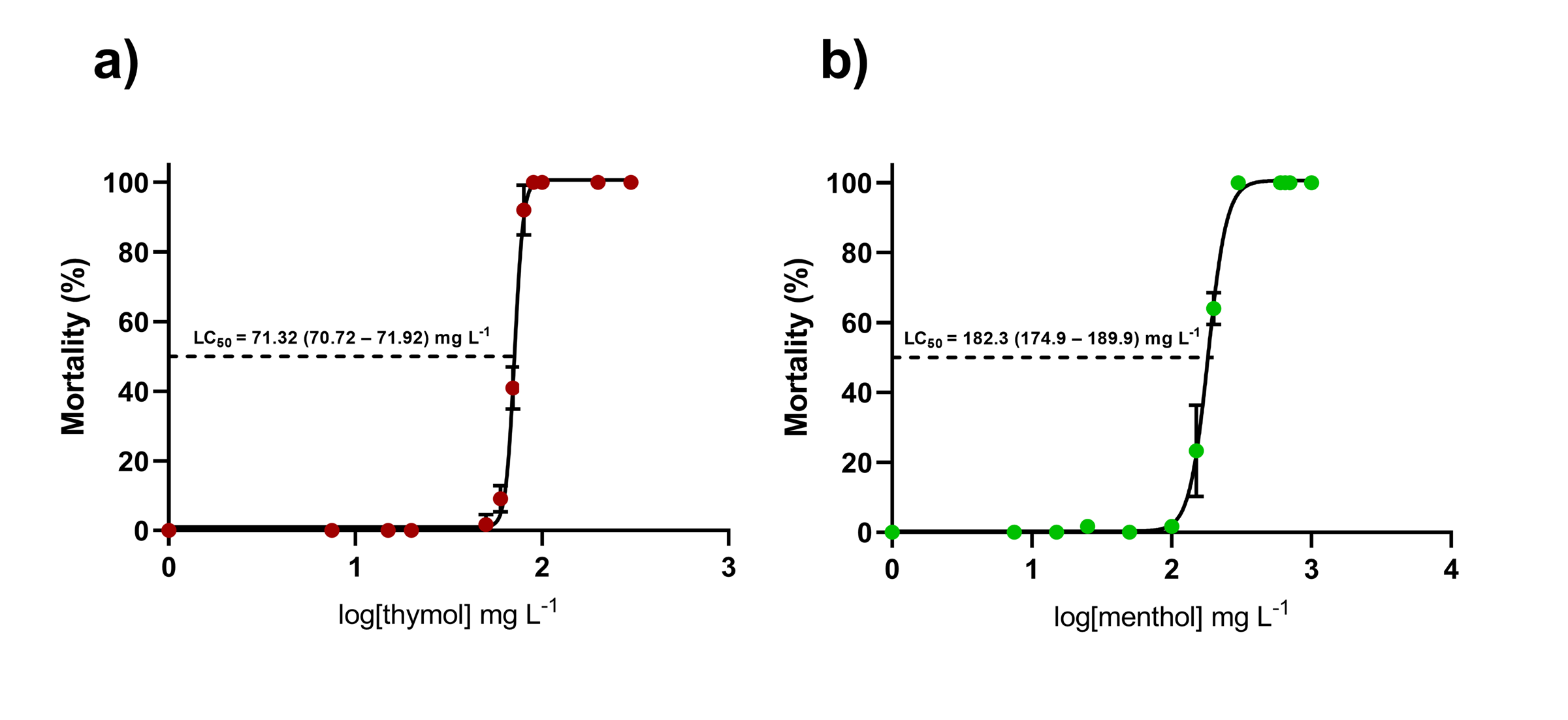

Supplement: Supplementary file 1 — (PNG 120 KB) [file 10695_2025_1530_Fig6_ESM.png]

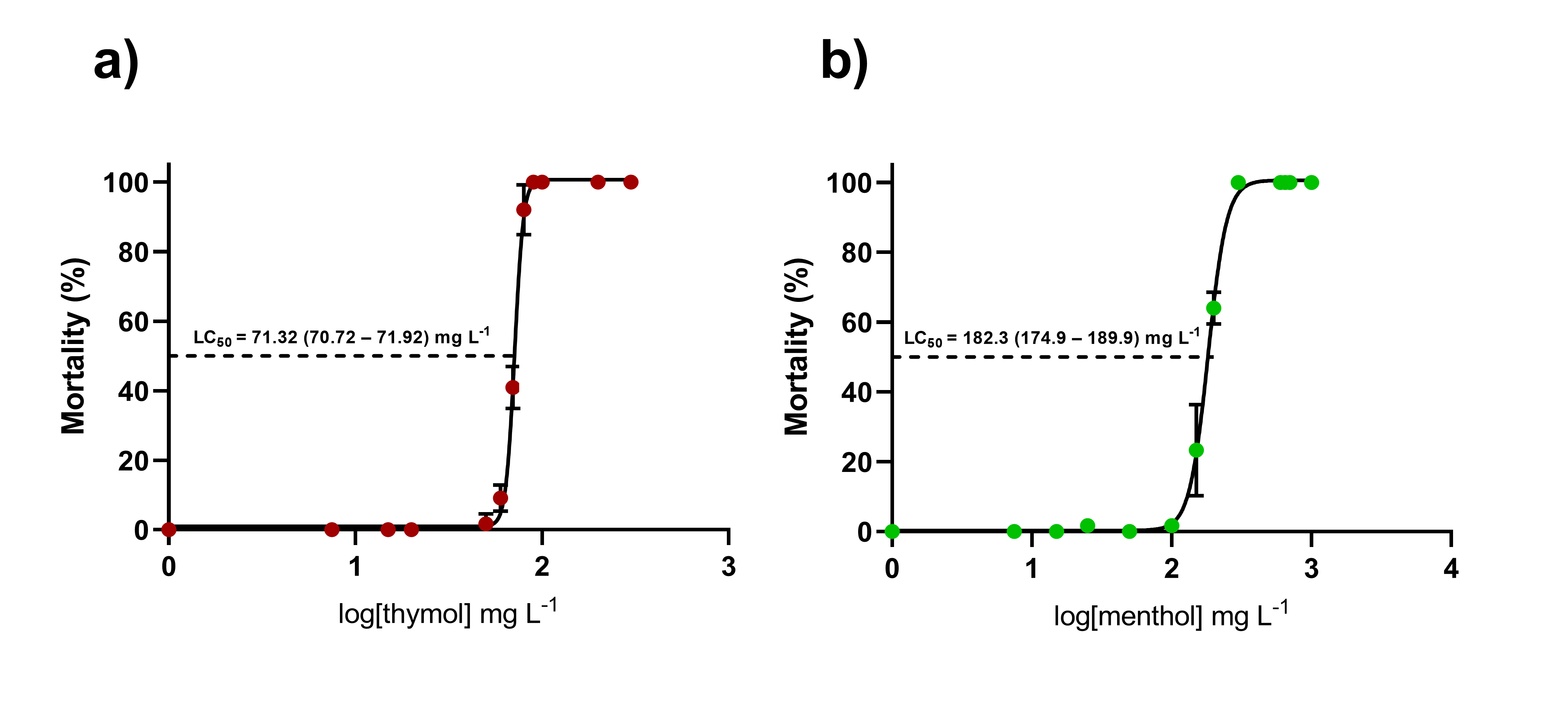

Supplement: Supplementary file 2 — High Resolution Image (TIF 604 KB) [file 10695_2025_1530_MOESM1_ESM.tif]
