# Supplementary material for: Thymol and menthol as anaesthetics for short transportation of zebrafish larva
Source: Fish Physiol Biochem. 2025 Jul 30;51(4):129. doi: 10.1007/s10695-025-01530-x (PMC12310772; doi:10.1007/s10695-025-01530-x)
Supplement: Supplementary file 3 — Supplementary file2 (DOCX 32 KB) [file 10695_2025_1530_MOESM2_ESM.docx]

**Table S1 - Descriptive statistics for physiological markers**

|  | | **Naïve** | **Control** | **t-value** | **p-value** | **Control** | **MS-222** | **E80** | **T15** | **M50** | **Statistical** | **p-value** |  |  |
| --- | --- | --- | --- | --- | --- | --- | --- | --- | --- | --- | --- | --- | --- | --- |
| **Heartbeat Rate** | | | | | | | | | | | | |  |  |
| **Heartbeat Rate**  (bpm – beat per minute) | 150.6 ± 8.0 | | 153.4 ± 6.3 | 0.6 | 0.6 | **153.4 ± 6.3^a^** | **137.8 ± 9.6^b^** | **137.6 ± 5.4^b^** | **122.0 ± 6.5^b^** | **114.6 ± 4.8^b^** | **F (4, 20) = 25.4** | **0.0001** |  |  |
| **Metabolic alterations** | | | | | | | | | | | | |  |  |
| **Metabolic rate** (AFU – arbitrary fluorescence units) | **4.5 ± 0.7** | | **12.3 ± 5.3*** | **3.3** | **0.01** | 12.3 ± 5.3 | 8.6 ± 1.1 | 13.0 ± 4.2 | 9.0 ± 2.1 | 12.3 ± 1.7 | F (4, 20) = 2.0 | 0.1 |  |  |
| **ATPase**  (mmol Pi/mg protein) | **7.2 ± 2.0** | | **1.9 ± 0.4*** | **5.9** | **0.0004** | **1.9 ± 0.4^a^** | **0.6 ± 0.4^b^** | **0.7 ± 0.4^b^** | **1.0 ± 0.4^b^** | **0.5 ± 0.2^b^** | **F (4, 20) = 11.2** | **0.0001** |  |  |
| LDH  (nmol NADH/min.mg protein) | 7.8 ± 1.6 | | 8.2 ± 2.9 | 0.3 | 0.8 | 8.2 ± 2.9 | 10.5 ± 1.6 | 9.9 ± 3.1 | 9.5 ± 2.9 | 10.8 ± 2.5 | F (4, 20) = 0.7 | 0.6 |  |  |
| **Stress parameters** | | | | | | | | | | | | | |  |
| Cortisol  (fg/mg protein) | 65.2 ± 21.2 | | 37.2 ± 16.4 | 2.2 | 0.06 | 37.2 ± 16.4 | 51.1 ± 26.8 | 48.5 ± 14.8 | 32.9 ± 13.7 | 61.9 ± 10.1 | F (4, 18) = 1.8 | 0.2 | | |
| **Glucose**  (mg glucose/mg protein) | **15.6 ± 2.2** | | **19.8 ± 3.0*** | **2.6** | **0.03** | **19.8 ± 3.0^a^** | **4.0 ± 2.3^b^** | **0.7 ± 0.6^b^** | **5.8 ± 4.7^b^** | **2.6 ± 1.7^b^** | **F (4, 19) = 33.8** | **0.0001** | | |
| **Lactate**  (mg lactate/mg protein) | 2.0 ± 0.5 | | 2.2 ± 0.6 | 0.4 | 0.7 | **2.2 ± 0.6^ab^** | **2.8 ± 1.5^ab^** | **0.7 ± 0.02^ab^** | **4.8 ± 2.0^b^** | **2.8 ± 1.3^ab^** | **F (4, 18) = 5.5** | **0.005** | | |

Data are expressed as mean ± SD for parametric data distribution. Statistical analysis was performed using Student’s t test (unpaired) to compare naïve group with control group, and used one-way ANOVA followed by Dunnett's multiple-comparison test for comparation of control group and anaesthetized groups. *Indicate significant differences between naïve and control group (p < 0.05) and different lowercase letters indicate significative differences between control group and anaesthetized groups (p < 0.05).

|  | **Naïve** | **Control** | **t-value** | **p-value** | **Control** | **MS-222** | **E80** | **T15** | **M50** | **Statistical** | **p-value** |
| --- | --- | --- | --- | --- | --- | --- | --- | --- | --- | --- | --- |
| **Oxidative stress** | | | | | | | | | | | |
| ROS  (µmol DCF/mg protein) | 551.7 ± 61.6 | 524.3 ± 56.3 | 0.7 | 0.5 | 524.3 ± 56.3 | 455.8 ± 51.0 | 499.2 ± 62.1 | 567.0 ± 202.7 | 679.4 ± 268.9 | F (4, 20) = 1.5 | 0.3 |
| **SOD**  (U/mg protein) | 101.3 ± 49.8 | 114.1 ± 40.1 | 0.5 | 0.7 | **114.1 ± 40.1^a^** | **247.7 ± 68.8^ab^** | **319.0 ± 131.3^b^** | **233.7 ± 99.2^ab^** | **265.8 ± 83.6^b^** | **F (4, 20) = 3.5** | **0.02** |
| **CAT**  (U/mg protein) | 17.9 ± 14.0 | 15.8 ± 7.8 | 0.3 | 0.8 | **15.8 ± 7.8^a^** | **4.7 ± 1.7^b^** | **3.5 ± 2.0^b^** | **5.6 ± 2.0^b^** | **2.4 ± 1.3^b^** | **F (4, 20) = 9.9** | **0.0001** |
| GPx  (nmol NADPH/min.mg protein) | 2.3 ± 1.2 | 1.9 ± 0.4 | 0.8 | 0.4 | 1.9 ± 0.4 | 3.1 ± 0.9 | 2.1 ± 1.3 | 1.5 ± 1.2 | 1.9 ± 1.0 | F (4, 20) = 1.8 | 0.2 |
| GR  (nmol NADPH/min.mg protein) | 1.6 ± 1.2 | 3.6 ± 1.6 | 2.3 | 0.05 | 3.6 ± 1.6 | 9.0 ± 3.4 | 5.4 ± 2.1 | 7.5 ± 3.3 | 7.0 ± 5.0 | F (4, 19) = 1.9 | 0.2 |
| **GST**  (µmol CDNB/min.mg protein) | **0.01 ± 0.001** | **0.003 ± 0.002** | **7.4** | **0.0002** | **0.003 ± 0.002**^a^ | **0.01± 0.008^ab^** | **0.02 ± 0.008^b^** | **0.008 ± 0.002^ab^** | **0.01 ± 0.008^b^** | **F (4, 20) = 4.4** | **0.01** |
| **GSH**  (µmol GSH/mg protein) | **116.6 ± 41.5** | **70.5 ± 15.5** | **2.3** | **0.05** | **70.5 ± 15.5**^a^ | **20.7 ± 4.5^b^** | **14.2 ± 11.4^b^** | **30.8 ± 12.6^b^** | **21.4 ± 14.1^b^** | **F (4, 20) = 17.1** | **0.0001** |
| **GSSG**  (µmol GSSG/mg protein) | 86.2 ± 14.6 | 66.2 ± 17.0 | 2.0 | 0.1 | **66.2 ± 17.0^a^** | **17.9 ± 4.1^b^** | **12.0 ± 11.5^b^** | **32.7 ± 18.6^b^** | **23.7 ± 19.0^b^** | **F (4, 20) = 9.9** | **0.0001** |
| ISO | 1.3 ± 0.3 | 1.1 ± 0.1 | 1.7 | 0.1 | 1.1 ± 0.1 | 1.2 ± 0.1 | 1.4 ± 0.4 | 1.0 ± 0.2 | 1.0 ± 0.3 | F (4, 20) = 2.3 | 0.1 |
| **LPO**  (µmol MDA/mg protein) | 34.9 ± 16.7 | 25.7 ± 1.7 | 1.1 | 0.3 | **25.7 ± 1.7^a^** | **18.5 ± 5.9^ab^** | **11.8 ± 7.8^b^** | **19.8 ± 5.7^ab^** | **11.45 ± 2.9^b^** | **F (4, 19) = 5.5** | **0.004** |
| PC  (nmol DNPH/min.mg protein) | 5.5 ± 1.5 | 5.5 ± 2.3 | 0.02 | 1.0 | 5.5 ± 2.3 | 8.9 ± 2.1 | 9.5 ± 5.9 | 6.2 ± 1.1 | 8.84 ± 2.4 | F (4, 20) = 1.6 | 0.2 |
| **NO**  (µmol NANO2/mg protein) | 6.3 ± 1.7 | 7.1 ± 1.1 | 0.8 | 0.4 | **7.1 ± 1.1^a^** | **13.1 ± 1.3^b^** | **12.4 ± 1.3^ab^** | **9.1 ± 2.7^ab^** | **14.2 ± 4.8^b^** | **F (4, 16) = 4.5** | **0.01** |
| **NRF2 levels** | | | | | | | | | | |  |
| **NRF2**  (fold of control) | 1.2 ± 0.6 | 3.1 ± 2.6 | 1.6 | 0.2 | **3.1 ± 2.6^a^** | **1.2 ± 0.7^ab^** | **2.4 ± 1.3^ab^** | **1.5 ± 0.7^ab^** | **7.4 ± 1.3^b^** | **F (4, 19) = 12.1** | **0.0001** |
| **Apoptosis and DNA damage** | | | | | | | | | | | |
| Casp 3  (µmol pNA/mg protein) | 5.5 ± 3.5 | 2.7 ± 0.7 | 1.6 | 0.2 | 2.7 ± 0.7 | 4.0 ± 0.6 | 4.5 ± 0.9 | 4.2 ± 2.0 | 4.2 ± 1.2 | F (4, 19) = 1.6 | 0.2 |
| Casp 9  (µmol pNA/mg protein) | 6.8 ± 2.0 | 4.3 ± 1.2 | 2.5 | 0.05 | 4.3 ± 1.2 | 3.4 ± 0.8 | 4.1 ± 2.1 | 3.3 ± 1.7 | 6.4 ± 6.0 | F (4, 20) = 0.9 | 0.5 |
| AIF  (fold of control) | 1.0 ± 0.3 | 1.1 ± 0.5 | 0.5 | 0.6 | 1.1 ± 0.5 | 0.8 ± 0.5 | 1.2 ± 0.8 | 0.9 ± 0.3 | 0.4 ± 0.2 | F (4, 17) = 1.3 | 0.3 |
| Caspase 3  (fold of control) | 1.2 ± 0.4 | 1.6 ± 1.4 | 0.6 | 0.6 | 1.6 ± 1.4 | 2.1 ± 0.8 | 5.5 ± 3.2 | 1.8 ± 0.6 | 5.5 ± 5.6 | F (4, 20) = 2.3 | 0.1 |
| Cell death  (CTCF - corrected total cell fluorescence) | 624.7 ± 261.1 | 967.3 ± 325.8 | 1.8 | 0.1 | 967.3 ± 325.8 | 1007 ± 71.1 | 992.4 ± 194.2 | 741.3 ± 61.0 | 893.9 ± 115.5 | **F (4, 20) = 1.8** | 0.2 |
| DNA Damage  (µg DNA SB/mg protein) | 8.5 ± 4.2 | 9.1 ± 4.6 | 0.2 | 0.8 | 9.1 ± 4.6 | 11.1 ± 3.1 | 14.0 ± 2.0 | 8.1 ± 2.7 | 10.0 ± 5.6 | F (4, 19) = 1.5 | 0.3 |
| **Inflammatory evaluation** | | | | | | | | | | | |
| Cell inflammation (neutrophils number) | 160.0 ± 30.0 | 173.0 ± 71.0 | 0.4 | 0.7 | 173.0 ± 71.0 | 153.0 ± 47.0 | 131.0 ± 22.0 | 134.0 ± 9.0 | 130.0 ± 24.0 | F (4, 18) = 0.9 | 0.5 |

**Table S2 - Descriptive statistics for biochemical markers**

Data are expressed as mean ± SD for parametric data distribution. Statistical analysis was performed using Student’s t test (unpaired) to compare naïve group with control group, and used one-way ANOVA followed by Dunnett's multiple-comparison test for comparation of control group and anaesthetized groups. *Indicate significant differences between naïve and control group (p < 0.05) and different lowercase letters indicate significative differences between control group and anaesthetized groups (p < 0.05).
